# Estimation of single-year-of-age counts of live births, fetal losses, abortions, and pregnant women for counties of Texas

**DOI:** 10.1186/s13104-017-2496-x

**Published:** 2017-05-08

**Authors:** Bismark Singh, Lauren Ancel Meyers

**Affiliations:** 1 Graduate Program in Operations Research and Industrial Engineering, The University of Texas at Austin, 204 E Dean Keeton St, Austin, TX 78705 USA; 2 Integrative Biology, The University of Texas at Austin, 1 University Station C0930, 78712 Austin, TX USA

**Keywords:** Live birth, Abortion, Fetal loss, Single-year-of-age, Pregnant women

## Abstract

**Objectives:**

We provide a methodology for estimating counts of single-year-of-age live-births, fetal-losses, abortions, and pregnant women from aggregated age-group counts. As a case study, we estimate counts for the 254 counties of Texas for the year 2010.

**Results:**

We use interpolation to estimate counts of live-births, fetal-losses, and abortions by women of each single-year-of-age for all Texas counties. We then use these counts to estimate the numbers of pregnant women for each single-year-of-age, which were previously available only in aggregate. To support public health policy and planning, we provide single-year-of-age estimates of live-births, fetal-losses, abortions, and pregnant women for all Texas counties in the year 2010, as well as the estimation method source code.

**Electronic supplementary material:**

The online version of this article (doi:10.1186/s13104-017-2496-x) contains supplementary material, which is available to authorized users.

## Background

Estimates of pregnant populations in a geographic region can be critical to assessing public health risks, such as chemical exposure [1], alcohol use [2], and advanced maternal age [3]. Such estimates have also informed teenage pregnancy prevention plans [4], the locations of abortion clinics [5, 6], and smoking ordinances [7]. However, the precision and effectiveness of such efforts have been limited by their reliance on aggregated rather than age-specific pregnancy counts.

Pregnant populations can be estimated from counts of live births, fetal losses and abortions. However, such data are often aggregated into 3–5 year age groups. Only a handful of studies provide single-year-of-age pregnancy estimates, including several addressing the prevalence of Down’s syndrome [8–10], fetal losses [11], and cross-age pregnancy comparisons [12].

Here, we describe an interpolation method for estimating single-year-of-age pregnancy counts from readily available, aggregated year-age live births, fetal losses and abortion data. To demonstrate the method, we derive county-level estimates across the state of Texas for the year 2010.

## Main text

### Nomenclature

We summarize the notation for our analysis below.

#### Sets

$$i \in I$$
set of counties
$$j \in J$$
set of single-year-of-age groups for which we estimate live birth/abortion/ fetal loss counts: $\{[10-11), [11-12), \ldots , [49-50)\}$
$$k \in K$$
set of aggregated year-age groups for which we have live birth/abortion/ fetal loss counts:
*Texas live births* $\{[10-15), [15-18), [18-20), [20-30), [30-40), [40-50)\}$
*Texas abortions* $$\{[10-15), [15-18), [18-20), [20-25), [25-30), [30-35), [35-40), [40-50)\}$$
*Texas fetal losses* $\{[10-15), [15-18), [18-20), [20-25), [25-30), [30-35), [35-40),[40-50)\}$
$$j \in J_k$$
   set of single-year age groups for which we estimate live birth/abortion/ fetal loss counts within the set of aggregated year-age group *k*

#### Data

$$w_{ij}$$
count of women in age group *j* in county *i*
$$B_{ik}$$
count of live births from women in age-group *k* in county *i*
$$A_{ik}$$
count of abortions from women in age-group *k* in county *i*
$$F_{k}$$
count of fetal-losses from women in age group *k* for entire country
$${TB}_j$$
live births from women in age-group *j* for entire state
$$p_b$$
proportion of year a woman is pregnant when she has a live birth: $\frac{9}{12}$
$$p_f$$
proportion of year a woman is pregnant when she has a fetal loss: $\frac{2}{12}$
$$p_a$$
proportion of year a woman is pregnant when she has an abortion: $\frac{3}{12}$

#### Parameters

$$b_{ij}$$
live births from women in age-group *j* in county *i*
$$f_{ij}$$
fetal losses from women in age-group *j* in county *i*
$$a_{ij}$$
abortions from women in age-group *j* in county *i*

We seek counts of live births, fetal losses, and abortions denoted by $$b_{ij}$$, $$f_{ij}$$, and $$a_{ij}$$, respectively for county *i*, at a single-year-of-age resolution ($$j \in J$$), but counts are only available in aggregated year-age resolution groups ($$k \in K$$). As an example, for our case study, we have counts of live births in a county from women of age-group $$k =[18-21)$$, but do not have counts of live births from women of age-groups $$j_1 = [18-19), \, j_2= [19-20)$$, and $$j_3=[20-21)$$. To obtain live births of a single-year-of-age, $$b_{ij}$$, we use a county-specific smoothed weighted interpolation scheme. We use aggregated year-age counts of live births, $$B_{ik}$$, available from the Texas Department of State Health Services (DSHS) [13], and derive weights from state-wide age-specific live birth information available from the Centers for Disease Control and Prevention (CDC) [14]. For abortions, $$a_{ij}$$, we use a county-specific cubic interpolation scheme. We use aggregated year-age counts of abortions, $$A_{ik}$$, available from the Texas DSHS [15]. For fetal losses, $$f_{ij}$$, we follow CDC recommendations [16] and use the same national fetal loss rate for all locations. We use a cubic Hermite interpolation scheme and use national aggregated year-age counts for fetal losses, $$F_k$$, available from Ventura et al. [17]. We provide details on these estimations in the proceeding sections.

Further, supplementary files for this subsection are provided in Additional files 1 and 2.

We also define a subset, $$J_k$$, of the set of single-year age groups, *J*, as a set of single-year age groups contained in the set *k*. For example, for $$k=[18-21)$$ we have $$J_k = \{ [18-19), [19-20), [20-21)\}$$. Finally, we assume that no woman is pregnant beyond the age of 50 and below the age of 10.

### Live births

**Fig. 1:**
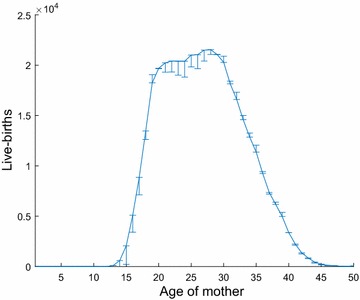
Counts of live births versus age of mother, ${TB}_j$, for Texas in 2010 and error in estimation, ${TB}_j-\sum _{i\in I}{b_{ij}}$

The National Vital Statistics System (NVSS) [14] provides counts for live births by single-year-of-age of the mother for the entire state of Texas, for the year 2010. We denote this quantity by ${TB}_j$, and present it in Fig. 1. However, the NVSS does not provide counts for live births by single-year-of-age of the mother, $b_{ij}$, for all counties of Texas for the year 2010. Further, aggregated year-age counts of live births, $B_{ik}$, are available from [13]. We describe our estimation scheme for $b_{ij}$ below.^1^

We assume all live births in a county for aggregated age-group, *k*, are proportional to the total live births in the entire state for that aggregated age-group, for all counties; i.e., $b_{ij} \propto {TB}_j, \forall j \in J_k, k \in K, i \in I$. With this assumption we can calculate values for $b_{ij}$.

**Fig. 2:**
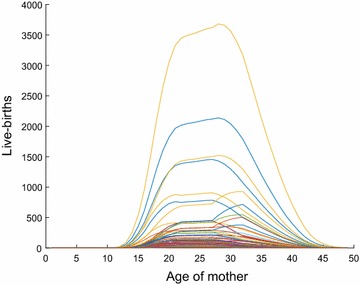
Counts of live births versus age of mother, $b_{ij}$, for the 254 counties in Texas in 2010

Under this assumption, we do not associate live births across one aggregated age-group to another. For example, we do relate the number of live births from a mother of age [21–22) to those of age [22–23), since they both belong to the set $J_{k_4}$, but we do not relate the number of births from a mother of age [29–30) to that of age [30–31) as they belong to $J_{k_4}$ and $J_{k_5}$, respectively. This can produce sharp changes in estimates of live births at the bin endpoints; i.e., at $j=J_{|k|}, \forall k \in K$. If this is undesired, we can use a moving-average filter to smooth out the estimates. Figure 2 plots the single-year estimates after the smoothing for the 254 counties of Texas. An alternative, is the stricter condition to assume all single-year-of-age live births are proportional to those in the entire state, for all counties; i.e., $b_{ij} \propto {TB}_j, \forall j \in J, i \in I$.

Further, the estimates for this subsection are provided in Additional files 3, 4 and 5.

### Fetal losses

The CDC recommends the use of national-average, as opposed to state-specific, fetal loss rates because most state reports of fetal deaths are limited to those with at least 20 weeks of gestation [16]; see also Macdorman et al. [18] and Ventura et al.  [17]. Despite many states having more current data than national aggregates, the national data is more accurate [16]. Limiting fetal loss reporting to at least 20 weeks of gestation could be a significant underestimate. The National Survey of Family Growth estimates about one million fetal losses per year in the United States, with majority of these occurring before the reporting requirements are met [17]. For more details on the accuracy of available fetal loss data versus gestation time [19].

**Fig. 3:**
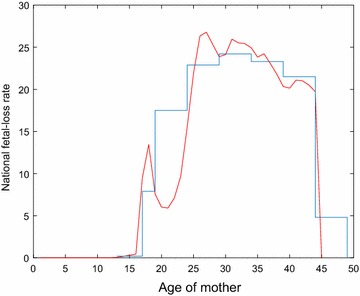
National fetal loss rate (number of fetal losses per 1000 women) versus age of mother. The* blue* steps are aggregated data. The *red curve* is our estimate for all points, using interpolation

As with live births, counts of fetal losses for single-year-of-age, $f_{ij}$, are not available. We seek to estimate these counts using the available year-aggregated fetal loss rate for age group *k*, from Ventura et al.  [17]. The fetal loss rates from Ventura et al. [17] are up to the year 2008, and we assume the rate did not change between 2008 and 2010. Since, the work in [17] does not report fetal losses from women aged 45 years and above, we assume no fetal losses occur in women above the age of 45. The blue steps in Fig. 3 present the aggregated national fetal loss rates; i.e., the number of fetal losses per 1000 women. Multiplying this rate by the national single-year-of-age counts of women, available from the US Census Bureau [20], and dividing by 1000, we obtain $F_k$ or the number of fetal losses for age group *k*.

**Fig. 4:**
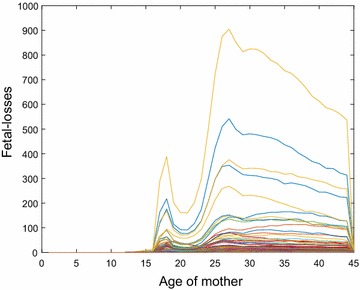
Counts of fetal losses versus age of mother, $f_{ij}$, for the 254 counties in Texas in 2010

Next, we use a piecewise-cubic Hermite interpolating polynomial (pchip) to determine single-year-of-age counts of national fetal losses.^2^ The national female population of age-group *j* is available from US Census Bureau [20]. The red curve in Fig. 3 plots the estimated national fetal loss rate (fetal losses per 1000 women) versus the age of the mother. Single-year-of-age counts of women in 2010, $w_{ij}$, for counties of Texas are also available from the US Census Bureau [20]. We, thus, obtain the number of fetal losses in a county *i* by multiplying $\frac{w_{ij}}{1000}$ with the previously obtained national fetal loss rate for age-group *j*. We present $f_{ij}$ in Fig. 4.

Further, the estimates for this subsection are provided in Additional files 6, 7, 8 and 9.

### Abortions

**Fig. 5:**
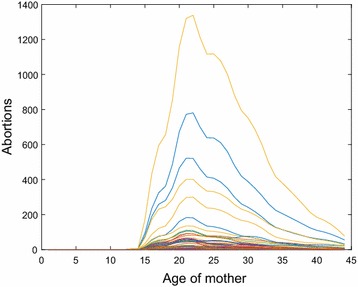
Counts of abortions versus age of mother, $a_{ij}$, for Texas in 2010

Unlike fetal losses, the CDC does not recommend the use of a national abortion rate due to wide variations in modes of data collection in geographic and demographic sub-populations [16]. County-specific counts of abortions in 2010 aggregated by mother’s age, $A_{ik}$, are available from Texas DSHS [15]. We use a pchip scheme almost identical to our procedure for estimating fetal losses (and thus do not present the details), but applied separately to each county. Figure 5 plots the distribution of counts of abortions, $a_{ij}$, for the 254 counties in Texas.

Further, the estimates for this subsection are provided in Additional files 10 and 11.

### Pregnant women

Finally, the number of pregnant women of age *j* for county *i* can be estimated as in [16],

1 $$\begin{aligned} b_{ij} p_b + f_{ij} p_f + a_{ij} p_a . \end{aligned}$$

**Fig. 6:**
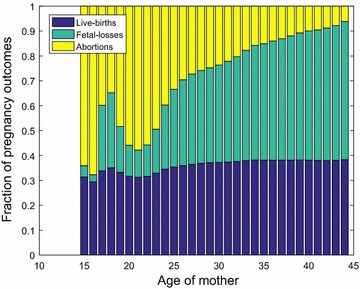
Fraction of pregnancies resulting in live births, fetal losses and abortions versus age of mother, for the 254 counties in Texas in 2010

Here, $b_{ij}, f_{ij}$, and $a_{ij}$ are estimated in the sections above. Figure 6 presents the fractional outcome of pregnancies for ages [15–44).

Further, the details for this subsection are provided in Additional file 12.

## Limitations

We make several comparisons between our estimates and reported data to assess the accuracy of our method. First, we compare our live birth estimates from age *j* as $\sum _{i}{b_{ij}}$, (using smoothed values of $b_{ij}$) to the known ${TB}_j$, and find an overall error of 3.84% (Fig. 1).

Second, the CDC reported a national fetal loss rate for women aged 15–44 in 2008 of 17.9 fetal losses per 1000 women [17], while our estimate for this age group in Texas is 18.0 fetal losses per 1000 women. However, the Texas DSHS [22] reported a much lower rate for women aged 15–44 of 0.46 fetal losses per 1000 women. This discrepancy likely stems from the Texas DSHS reporting criteria. DSHS tracks only fetal losses occurring after 20 weeks of gestation or with a birth weight of at least 500 g [23], while almost three quarters of fetal losses occur in the first trimester [16]. Across the US, state reporting requirements [23, 24] and reporting regularity [25] vary considerably. We support the CDC recommendations to assume national fetal loss estimates for county-level assessments. Our estimate of 16.9% fetal losses among all pregnancies in 2010 is consistent with the CDC report that approximately one in six pregnancies ended in a fetal loss in 2004 [16].

Finally, our estimate of 73,481 abortions among Texas residents in 2010 is close to the Texas DSHS report of 73,600 [22], yielding an error of 0.16%. Our method does not consider Texas residents who received abortions outside the state, which can be significant [26]. Estimates that include out of state abortions are slightly higher–75,151 reported by the CDC’s Abortion Surveillance System [27] and 79,390 reported by Jones et al. [28].

## Additional files

**Additional file 1.** This file contains the source information of all the input files we provide.

**Additional file 2.** This file contains a summary of all the input and output files we provide.

**Additional file 3.** This file contains aggregated live births for the 254 counties of Texas for the year 2010.

**Additional file 4.** This file contains single-year-of-age live births for the entire state of Texas.

**Additional file 5.** This file contains our estimated single-year-of-age live births for the 254 counties of Texas for the year 2010.

**Additional file 6.** This file contains single-year-of-age national populations of women for the year 2010.

**Additional file 7.** This file contains national aggregated fetal loss rates and corresponding populations of women for the year 2008.

**Additional file 8.** This file contains single-year-of-age populations of women for the 254 counties of Texas for the year 2010.

**Additional file 9.** This file contains our estimated single-year-of-age fetal losses for the 254 counties of Texas for the year 2010.

**Additional file 10.** This file contains aggregated abortions for the 254 counties of Texas for the year 2010.

**Additional file 11.** This file contains our estimated single-year-of-age abortions for the 254 counties of Texas for the year 2010.

**Additional file 12.** This file contains our estimated single-year-of-age fractional pregnancy outcomes for the entire state of of Texas for the year 2010.

## Footnotes

1 We have: ${TB}_j = \sum _{i \in I} {b_{ij}} , \forall j \in J$, and $\sum _{j \in J_k} {b_{ij}} = B_{ik} , \forall i \in I, \forall k \in K$. This gives a set a of $|I||K| + |J|$ equations with |*I*||*J*| unknowns. Further, |*K*| equations are redundant. Since, $|K| < |J|$ and $|I|>1$, the system of equations is undetermined.

2 Other interpolation methods include linear interpolation and spline. Spline interpolation is known to produce smoother outputs, which can prevent the need for smoothing as a secondary step, but pchip prevents overshoots at intermediate points. For an introduction to the relative merits of different interpolation methods see, e.g., [21].
